# Evaluation of a Text Message–Based COVID-19 Vaccine Outreach Program Among Older Patients: Cross-sectional Study

**DOI:** 10.2196/33260

**Published:** 2022-07-18

**Authors:** Naheed Ahmed, Christian Boxley, Ram Dixit, Seth Krevat, Allan Fong, Raj M Ratwani, Deliya B Wesley

**Affiliations:** 1 NYU Grossman School of Medicine New York, NY United States; 2 MedStar Center for Health Equity Research Hyattsville, MD United States; 3 MedStar Health National Center for Human Factors in Healthcare Hyattsville, MD United States; 4 Mathematica Washington, DC United States

**Keywords:** vaccine outreach, text messaging, elderly patients, evaluation, smartphone, text message, SMS, appointment, elderly, older adults, vaccine, effectiveness, engagement, cross-sectional

## Abstract

**Background:**

COVID-19 vaccines are vital tools in the defense against infection and serious disease due to SARS-CoV-2. There are many challenges to implementing mass vaccination campaigns for large, diverse populations from crafting vaccine promotion messages to reaching individuals in a timely and effective manner. During this unprecedented period, with COVID-19 mass vaccination campaigns essential for protecting vulnerable patient populations and attaining herd immunity, health care systems were faced with the dual challenges of vaccine outreach and distribution.

**Objective:**

The aim of this cross-sectional study was to assess the effectiveness of a COVID-19 vaccine text outreach approach for patients aged 65 years and older. Our goal was to determine whether this approach was successful in scheduling patients for COVID-19 vaccine appointments.

**Methods:**

We developed SMS text messages using the Tavoca platform. These messages informed patients of their vaccine eligibility and allowed them to indicate their interest in scheduling an appointment via a specific method (email or phone) or indicate their lack of interest in the vaccine. We tracked the status of these messages and how patients responded. Messages were sent to patients aged 65 years and older (N=30,826) at a nonprofit health care system in Washington, DC. Data were collected and examined from January 14 to May 10, 2021. Data were analyzed using multivariate multinomial and binary logistic regression models in SAS (version 9.4; SAS Institute Inc).

**Results:**

Approximately 57% of text messages were delivered to patients, but many messages received no response from patients (40%). Additionally, 42.1% (12,978/30,826) of messages were not delivered. Of the patients who expressed interest in the vaccine (2938/30,826, 9.5%), Black or African American patients preferred a phone call rather than an email for scheduling their appointment (odds ratio [OR] 1.69, 95% CI 1.29-2.21) compared to White patients. Patients aged 70-74 years were more likely to schedule an appointment (OR 1.38, 95% CI 1.01-1.89) than those aged 65-69 years, and Black or African American patients were more likely to schedule an appointment (OR 2.90, 95% CI 1.72-4.91) than White patients.

**Conclusions:**

This study provides insights into some advantages and challenges of using a text messaging vaccine outreach for patients aged 65 years and older. Lessons learned from this vaccine campaign underscore the importance of using multiple outreach methods and sharing of patient vaccination status between health systems, along with a patient-centered approach to address vaccine hesitancy and access issues.

## Introduction

Authorization of the emergency use of COVID-19 vaccines, which were developed and tested in record time, was a seminal moment in efforts to control the COVID-19 pandemic [1]. These vaccines are vital tools in the prevention of infection and serious disease from SAS-CoV-2, and COVID-19 mass vaccination campaigns have been essential for protecting vulnerable patient populations and achieving herd immunity. Launching these vaccination campaigns has been challenging for several reasons related to logistical issues of producing, storing, and transporting vaccines, and patient-level factors such as vaccine access and vaccine hesitancy [2-5]. Health care systems were starting points for vaccination campaigns given their direct access to patient populations and having the personnel and resources to store vaccines and vaccinate patients. However, the COVID-19 vaccination effort was unprecedented in its scale, and many health care systems across the United States were faced with challenges of outreach, equity, scheduling, and administration.

With no blueprint for this type of vaccination campaign, health care systems—particularly those with large diverse patient populations—had to quickly design and launch outreach efforts to patients [2]. These efforts were further complicated by ongoing challenges with patient distrust of medical institutions, vaccine hesitancy and access barriers, and interoperability of health record databases [3,6]. Outreach to patients was complicated by out-of-date or incorrect demographic information, which was necessary for determining vaccine eligibility of each patient. Other complications included the absence of coordination between adjacent health care systems, as patients had the opportunity to be vaccinated at other health care sites, but these vaccination records were not shared among health care systems.

Reflecting on these challenges, this cross-sectional study used a not-for-profit health system as a case study to examine the intersection of health information technology and health disparities in vaccine outreach efforts. The focus of this study was on patients aged 65 years and older as they were among the first to be eligible for the COVID-19 vaccine. Additionally, patients in this age group may face additional challenges to vaccination, such as complicated medical conditions, transportation needs, and reliance on caregivers to assist with medical decision-making [7]. Findings from this study generated important lessons learned for ongoing efforts to increase COVID-19 vaccination rates among patients aged 65 years and older.

## Methods

### Study Site

This study took place in a not-for-profit health system with 10 hospitals, over 280 outpatient clinics, and nearly 2 million patients in the mid-Atlantic region of the United States.

### Target Population

**Figure 1:**
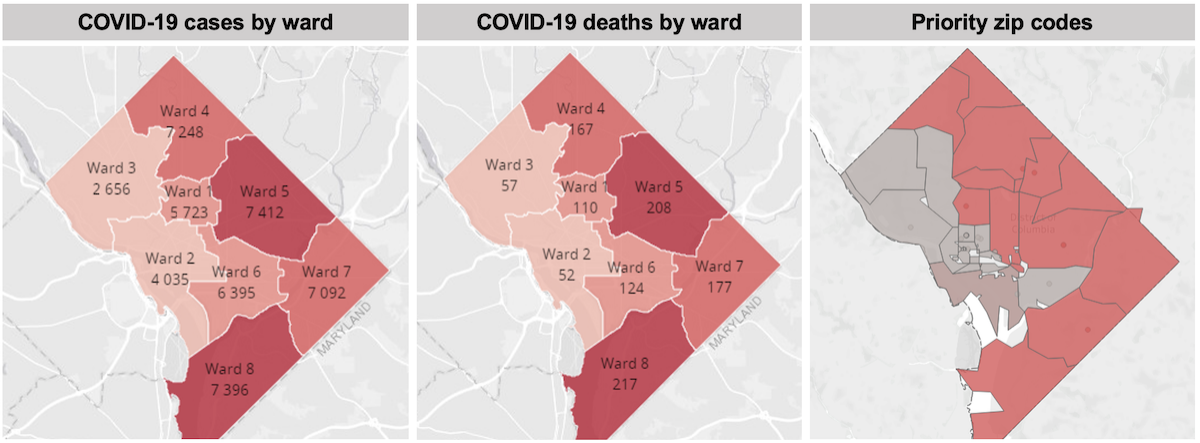
Maps of COVID-19 cases and deaths by ward and priority zip codes for vaccination.

For this study, COVID-19 vaccine outreach to patients in the Washington, DC catchment area was examined. In Washington, DC, the local health department prioritized patients aged 65 years and older in the initial rollout of the vaccine to the public [8,9]. Additionally, certain zip codes were prioritized for outreach efforts based on the disproportionate impact of COVID-19 in terms of morbidity and mortality rates. A side-by-side comparison of priority zip codes with COVID-19 cases and deaths is presented in Figure 1.

Similar to nationwide trends, COVID-19 cases and deaths were concentrated in areas with greater proportions of racial or ethnic minorities and low-socioeconomic-status populations in Washington, DC. Four health systems in the region, the local health department, and local community organizations (eg, faith-based and nonprofit organizations) conducted outreach to residents in priority zip codes, including virtual webinars and door-to-door canvassing for the purpose of answering questions and concerns about the COVID-19 vaccines [10].

### Study Population

The health system serves approximately 45,000 Washington, DC residents aged 65 years and older, which required a large-scale outreach operation to contact, screen, and schedule these patients for vaccination. It is important to note that local policy at the time permitted health systems to only vaccinate patients who had previously been served by the health system. Information on whether a patient was vaccinated at a different facility was unavailable.

### Inclusion and Exclusion Criteria

Eligibility was limited to patients aged 65 years and older with a Washington, DC address per local health department vaccination guidelines. Patients who had been seen at any of the health system facilities in the last 5 years were contacted about the COVID-19 vaccine.

### Text Message Vaccine Outreach

**Figure 2:**
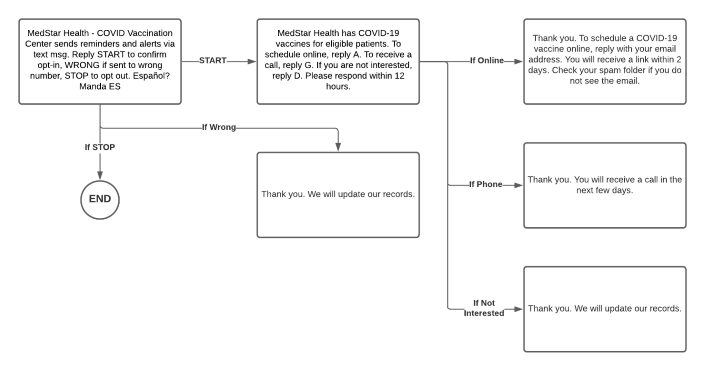
Text messaging flow.

During the initial phase of outreach, a dedicated call center contacted patients who met vaccine eligibility criteria. Operators were only able to speak to 41% (1093/2670) of patients contacted, and only 42% (458/1093) of connected calls resulted in scheduled appointments. Thus, only 17% of total calls resulted in a scheduled appointment. Given the relatively slow pace and resource-intensiveness of this approach, a transition was made to an automated text messaging and follow-up strategy, in which patients were sent a text message informing them that they were eligible to receive the vaccine and asking if they were interested in scheduling an appointment. The text messaging platform, Tavoca, was used to create message templates informing patients of their vaccine eligibility and allowing them to indicate their interest in scheduling via a specific method (email or phone) or indicate their lack of interest in the vaccine. Text messages were drafted on the basis of character limits of the Tavoca platform, and the text sequence is shown in Figure 2.

Batches of text messages were sent on the basis of a prioritization algorithm that ranked patients by age and priority zip code, starting with the oldest patients in priority zip codes and then patients in nonpriority zip codes. Patients who indicated they were interested in the vaccine were asked which communication platform they preferred (phone or email), and depending on their response, they were referred to a call center or a web-based scheduling system. There was a high volume of messages not delivered owing to phone numbers no longer being in operation and some being landlines, which do not accept text messages.

### Data Collection and Management

Patient information was extracted from the electronic health record and scheduling systems. Data were extracted and analyzed from January 14 to May 10, 2021. During this stage of the vaccine rollout, only the Pfizer and Moderna COVID-19 vaccines were available to patients. For this analysis, patients who shared the same phone number (n=14,419) were excluded, as there was no way to accurately determine which of the multiple patients associated with one phone number responded to the text message (Table 1). Confirmed text messaging interaction data were available for 30,826 patients who had unique phone numbers, which were analyzed for this study (Table 1). The demographic characteristics of the excluded patients were similar to those of the patients included in the study sample.

**Table 1:** Demographic Information for excluded patients (N=14,419) and study participants (N=30,826).

Characteristics			Excluded patients, n (%)		Study participants, n (%)
**Sex**					
	Female	8224 (57.0)		18,399 (59.7)	
	Male	6181 (42.8)		12,427 (40.3)	
**Residence**					
	Priority zip code	11,408 (79.1)		24,211 (78.5)	
	Nonpriority zip code	3011 (20.8)		6615 (21.5)	
**Race or ethnicity**					
	Black or African American	8939 (61.9)		19,372 (62.8)	
	Hispanic	477 (3.31)		706 (2.3)	
	Asian	150 (1.04)		208 (0.67)	
	White	2885 (20.0)		7315 (23.7)	
	Other	1968 (13.6)		3225 (10.5)	
**Age (years)**					
	65-69	3506 (24.3)		9804 (31.8)	
	70-74	3186 (22.1)		7770 (25.2)	
	75-79	2527 (17.5)		5151 (16.7)	
	80-84	2092 (14.5)		3504 (11.4)	
	85-90	1505 (10.4)		2273 (7.4)	
	>90	1603 (11.1)		2324 (7.5)	
**Dose 1 received**					
	Yes	744 (5.1)		239 (0.77)	
	No	13,675 (94.8)		30,587 (99.2)	
**Dose 2 received**					
	Yes	643 (4.4)		232 (0.75)	
	No	13,776 (95.5)		30,594 (99.2)	

### Independent Variables

A total of 7 independent variables related to patient demographics and health system utilization in this study. Demographic variables included residing in a priority zip code (priority=1 and nonpriority=0), binary variables for each racial or ethnic group (Black or African American, Hispanic, Asian, White, and other), binary variables for each age group (65-69 years, 70-74 years, 75-79 years, 80-84 years, 85-89 years, and >90 years), and sex (male=1, female=0). Health system utilization variables included having a primary care provider (yes=1, no=0) and total visits in the past 5 years (continuous). A series of interaction variables were created for priority zip code and age and for priority zip codes and race or ethnicity.

### Outcome Variables

There were 4 primary outcome variables of interest. The first outcome variable was the status of the initial text message sent to patients: 1=delivered and interested in vaccine; 2=delivered and no response; 3=delivered and not interested in the vaccine; and 4=not delivered (number belongs to a landline or is no longer in operation). Among patients who expressed interest in the COVID-19 vaccine, the preferred communication method for scheduling the vaccine appointment (phone=1, email=0) and if an appointment was scheduled (scheduled=1, not scheduled=0) were assessed.

### Statistical Analysis

First, descriptive statistics were assessed to determine sample characteristics. Next, multivariate regression models were used to assess the association between patient demographics (priority zip codes, race or ethnicity, age, and sex) and outcomes of interest. Multivariate multinomial regression models were used for the first outcome of interest (text message status) and multivariate logistic regression for the other outcomes (preferred communication method and appointment scheduled). It was not possible to analyze vaccine uptake in this study owing to sample size limitations. Data were analyzed using SAS (version 9.4; SAS Institute Inc).

### Ethics Approval

This study received approval from the institutional review board of MedStar Health Research Institute (STUDY00002197).

## Results

### Sample Characteristics

Demographic information on study patients is detailed in Multimedia Appendix 1. The majority of patients (24,211/30,826, 78.5%) live in a priority zip code, which implies that there is high COVID-19 morbidity and mortality, 59.7% (18,399/30,826) of participants are female, and 62.8% (19,372/30,826) of participants are Black or African American. Further, 57% (17,848/30,826) of text messages were delivered to patients and 42.1% (12,978/30,826) of messages were not delivered (number belongs to a landline or is no longer in operation). Of the messages that were delivered, 40% (12,333/17,848) received no response. A few patients (2,938/17,848, 9.5%) expressed interest in getting the COVID-19 vaccine. Among patients who expressed interest in the vaccine, only 253 scheduled an appointment. A majority of patients who scheduled an appointment completed their first (214/226, 87.5%) and second (207/226, 80%) vaccine doses.

### Regression Models

First, differences among the 4 status options for the initial text message sent to patients were examined: interested in the vaccine, not interested in the vaccine, no response, and text message not delivered (Table 2). Notable findings include patients residing in priority zip codes who were more likely to “not respond” (odds ratio [OR] 1.19, 95% CI 1.12-1.27) than for message “not delivered” in comparison with patients living in nonpriority zip codes. In models that included age, among patients aged 70 years and older, the message was more likely to not be delivered than for there to be patient engagement with the message. Black or African American patients were less likely to be interested rather than not having received the message in comparison to White patients. Asian and Hispanic patients were more likely to not respond rather than not having received the message in comparison to White patients. Patients with a primary care provider were more likely to be interested rather than not having received the message in comparison to patients without a primary care provider.

Next, the preferred communication platform for scheduling an appointment among patients interested in the vaccine was assessed (Table 3). Patients living in priority zip codes were more likely to prefer a phone call (OR 1.43, 95% CI 1.15-1.78) than those living in a nonpriority zip code. These results remained the same with the addition of other independent variables in the models. Preference for a phone call was more likely among patients aged 70-74 years (OR 1.33, 95% CI 1.08-1.64) and those aged 80-84 years (OR 1.82, 95% CI 1.17-2.83) than among those aged 65-69 years, and among Black or African American patients (OR 1.69, 95% CI 1.29-2.21) than among White patients. Regarding the interaction effects, patients living in priority zip codes and those aged 75-79 years were significantly less likely to prefer a phone call (OR 0.40, 95% CI 0.20-0.81) than those aged 65-69 years and living in a priority zip code.

Finally, scheduled visits among patients who expressed an interest in being vaccinated were examined (Table 4). Patients aged 70-74 years were more likely to schedule an appointment (OR 1.38, 95% CI 1.01-1.89) than those aged 65-69 years, and Black or African American patients were more likely to schedule an appointment (OR 2.90, 95% CI 1.72-4.91) than White patients. Interaction variables could not be examined in these models given the small sample size.

**Table 2:** Odds ratios (ORs) of text status (N=30,826).

Text status			Model 1				Model 2					Model 3		
			OR		95% CI		OR		95% CI		OR			95% CI
**Living in a priority zip code**														
	Interested	0.83^a^		0.75-0.91		0.81^a^		0.73-0.89		0.90			0.80-01.01	
	No response	1.19^a^		1.12-1.27		1.17^a^		1.09-1.25		1.08			1.00-1.17	
	Not interested	0.48^a^		0.44-0.53		0.48^a^		0.43-0.52		0.97			0.86-1.09	
	Not delivered	Reference		Reference		Reference		Reference		Reference			Reference	
**Age groups (years)**														
	65-69	—^b^		—		Reference		Reference		Reference			Reference	
	70-74: interested	—		—		0.53^a^		0.48-0.59		0.53^a^			0.48-0.59	
	70-74: no response	—		—		0.51^a^		0.47-0.55		0.52^a^			0.48-0.56	
	70-74: not interested	—		—		0.66^a^		0.60-0.74		0.64^a^			0.57-0.72	
	70-74: not delivered	—		—		Reference		Reference		Reference			Reference	
	75-79: interested	—		—		0.25^a^		0.22-0.29		0.24^a^			0.21-0.28	
	75-79: no response	—		—		0.30^a^		0.28-0.32		0.31^a^			0.28-0.33	
	75-79: not interested	—		—		0.36^a^		0.32-0.41		0.33^a^			0.29-0.38	
	75-79: not delivered	—		—		Reference		Reference		Reference			Reference	
	80-84: interested	—		—		0.12^a^		0.10-0.15		0.12^a^			0.10-0.15	
	80-84: no response	—		—		0.20^a^		0.18-0.22		0.21^a^			0.19-0.23	
	80-84: not interested	—		—		0.16^a^		0.14-0.20		0.16^a^			0.13-0.19	
	80-84: not delivered	—		—		Reference		Reference		Reference			Reference	
	85-89: interested	—		—		0.06^a^		0.05-0.08		0.06^a^			0.05-0.08	
	85-89: no response	—		—		0.10^a^		0.09-0.12		0.11^a^			0.10-0.12	
	85-89: not interested	—		—		0.07^a^		0.06-0.10		0.08^a^			0.06-0.10	
	85-89: not delivered	—		—		Reference		Reference		Reference			Reference	
	>90: interested	—		—		0.03^a^		0.02-0.04		0.03^a^			0.02-0.05	
	>90: no response	—		—		0.07^a^		0.06-0.08		0.08^a^			0.07-0.09	
	>90: not interested	—		—		0.04^a^		0.03-0.06		0.04^a^			0.03-0.06	
	>90: not delivered	—		—		Reference		Reference		Reference			Reference	
**Sex**														
	Interested	—		—		—		—		1.48^a^			1.35-1.61	
	No response	—		—		—		—		2.11^a^			2.00-2.23	
	Not interested	—		—		—		—		1.36^a^			1.24-1.49	
	Not delivered	—		—		—		—		Reference			Reference	
**Race and ethnicity**														
	White	—		—		—		—		Reference			Reference	
	Black or African American: interested	—		—		—		—		0.80^a^			0.71-0.90	
	Black or African American: no response	—		—		—		—		1.31^a^			1.21-1.42	
	Black or African American: not interested	—		—		—		—		0.27^a^			0.24-0.31	
	Black or African American: not delivered	—		—		—		—		Reference			Reference	
	Asian: interested	—		—		—		—		1.58			0.96-2.61	
	Asian: no response	—		—		—		—		2.32^a^			1.64-3.38	
	Asian: not interested	—		—		—		—		0.85			0.50-1.43	
	Asian: not delivered	—		—		—		—		Reference			Reference	
	Hispanic or Latino: interested	—		—		—		—		1.10			0.75-1.62	
	Hispanic or Latino: no response	—		—		—		—		4.56^a^			3.70-5.61	
	Hispanic or Latino: not interested	—		—		—		—		0.59^a^			0.39-0.88	
	Hispanic or Latino: not delivered	—		—		—		—		Reference			Reference	
	Other race: interested	—		—		—		—		1.14			0.97-1.34	
	Other race: no response	—		—		—		—		1.75^a^			1.57-1.95	
	Other race: not interested	—		—		—		—		0.59^a^			0.51-0.70	
	Other race: not delivered	—		—		—		—		Reference			Reference	
	Total encounters: interested	—		—		—		—		1.00			1.00-1.01	
	Total encounters: no response	—		—		—		—		1.00			1.00-1.00	
	Total encounters: no response	—		—		—		—		1.00			1.00-1.00	
	Total encounters: not delivered	—		—		—		—		Reference			Reference	
	primary care provider: interested	—		—		—		—		1.17^a^			1.04-1.32	
	primary care provider: no response	—		—		—		—		0.75^a^			0.70-0.81	
	primary care provider: not interested	—		—		—		—		1.31^a^			1.16-1.49	
	primary care provider: not delivered	—		—		—		—		Reference			Reference	

^a^Significant at *P*<.05.

^b^Variable not used in model.

**Table 3:** Odds ratios (ORs) for the preferred communication platform: phone or email (n=2011).

		Model 1		Model 2		Model 3		Model 4		Model 5	
		OR	95% CI	OR	95% CI	OR	95% CI	OR	95% CI	OR	95% CI
Living in a priority zip code		1.43^a^	1.15-1.78	1.45^a^	1.16-1.81	1.06	0.82-1.38	—^b^	—	—	—
**Age groups (years)**											
	65-69	—	—	Reference	Reference	Reference	Reference	—	—	—	—
	70-74	—	—	1.33^a^	1.08-1.64	1.32^a^	1.07-1.63	—	—	—	—
	75-79	—	—	1.24	.93-1.64	1.26	.95-1.69	—	—	—	—
	80-84	—	—	1.82^a^	1.17-2.83	1.95^a^	1.25-3.04	—	—	—	—
	85-89	—	—	0.84	0.45-1.56	0.89	0.48-1.66	—	—	—	—
	>90	—	—	1.29	0.64-2.62	1.28	0.63-2.61	—	—	—	—
Sex		—	—	—	—	0.86	0.71-1.04	—	—	—	—
**Race and ethnicity**											
	White	—	—	—	—	Reference	Reference	—	—	—	—
	Black or African American	—	—	—	—	1.69^a^	1.29-2.21	—	—	—	—
	Asian	—	—	—	—	0.67	0.27-1.68	—	—	—	—
	Hispanic or Latino	—	—	—	—	0.94	0.41-2.15	—	—	—	—
	Other race	—	—	—	—	1.30	0.91-1.85	—	—	—	—
Total encounters		—	—	—	—	1.00	0.99-1.00	—	—	—	—
Has a primary care provider		—	—	—	—	1.29	0.99-1.70	—	—	—	—
**Interaction variables**											
	Living in a priority zip code and age group 65-69 years	—	—	—	—	—	—	Reference	Reference	—	—
	Living in a priority zip code and age group 70-74 years	—	—	—	—	—	—	0.69	0.41-1.15	—	—
	Living in a priority zip code and age group 75-79 years	—	—	—	—	—	—	0.40^a^	0.20-0.81	—	—
	Living in a priority zip code and age group 80-84 years	—	—	—	—	—	—	0.92	0.34-2.50	—	—
	Living in a priority zip code and age group 85-89 years	—	—	—	—	—	—	4.6	0.49-43.93	—	—
	Living in a priority zip code and age group >90 years	—	—	—	—	—	—	1.58	0.22-11.39	—	—
	Living in a priority zip code and White	—	—	—	—	—	—	—	—	Reference	Reference
	Living in a priority zip code and Asian	—	—	—	—	—	—	—	—	0.97	0.15-6.28
	Living in a priority zip code and Black or African American	—	—	—	—	—	—	—	—	1.00	0.55-1.82
	Living in a priority zip code and Hispanic	—	—	—	—	—	—	—	—	0.85	0.09-7.52
	Living in a priority zip code and other race	—	—	—	—	—	—	—	—	1.03	0.50-2.09

^a^Significant at *P*<.05.

^b^Variable not used in model.

**Table 4:** Odds ratios (ORs) of scheduled visits (n=2011).

			Model 1					Model 2					Model 3		
			OR		95% CI		OR			95% CI		OR			95% CI
Living in a priority zip code			1.30		0.90-1.87		1.29			0.89-1.87		0.72			0.46-1.11
**Age group (years)**															
	65-69	—^a^		—		Reference			Reference		Reference			Reference	
	70-74	—		—		1.38^b^			1.01-1.89		1.36			0.99-1.86	
	75-79	—		—		1.15			0.74-1.78		1.20			0.77-1.88	
	80-84	—		—		0.45			0.18-1.15		0.50			0.19-1.28	
	85-89	—		—		0.42			0.10-1.78		0.46			0.11-1.98	
	>90	—		—		2.25			0.95-5.31		2.36			0.98-5.67	
Sex			—		—		—			—		1.01			0.75-1.36
**Race and ethnicity**															
	White	—		—		—			—		Reference			Reference	
	Black or African American	—		—		—			—		2.90^b^			1.72-4.91	
	Asian	—		—		—			—		0.91			0.11-7.20	
	Hispanic or Latino	—		—		—			—		0.78			0.10-6.20	
	Other race	—		—		—			—		1.86			0.93-3.70	
Total encounters			—		—		—			—		1.00			1.00-1.00
Has a primary care provider			—		—		—			—		1.65			0.97-2.83

^a^Variable not used in model.

^b^Significant at *P*<.05.

## Discussion

### Principal Findings

Our findings highlight important considerations for large health systems attempting to use text messaging for effective and efficient vaccination outreach for diverse patient populations. Many text messages were not delivered (12,978/30,826, 42.1%) and of the messages that were delivered, 40% received no response. A small percentage of patients expressed interested in the vaccine (2938/30,826, 9.5%). Among patients who expressed interest in being vaccinated, it was found that patients largely preferred a phone call over email for scheduling their vaccine appointment. Of the patients contacted to schedule an appointment, 70-74–year-old patients were more likely to schedule an appointment than 65-69–year-old patients, and Black or African American patients were more likely to schedule an appointment than White patients.

These results reflect several key challenges. The first challenge was the verification of patient information and ascertaining whether a listed number was a landline or mobile number. Given the large number of text messages not delivered (n=12,978), a mechanism to validate these numbers is needed. In addition, a mechanism to update and clearly designate a number as a mobile number in the electronic health record is critical. Second, based on the selected outreach preference (phone) by those who were successfully contacted, it is critical to leverage nontext outreach methods for effective engagement of some populations. This finding may reflect comfort levels with technology among patients aged 65 years and older related to using a phone or computer to schedule an appointment. As patients had opportunities to be vaccinated elsewhere, it would be imprudent to draw any conclusions about why certain patient groups were more likely to schedule an appointment than others.

### Comparison With Other Studies

Results from another study that examined a COVID-19 vaccination text message outreach to older patients found that reminder messages and messages that instilled ownership in patients led to increased scheduling of appointments and vaccination rates [10]. The findings from this study reflect how messaging protocols and how messages are written influence patient responsiveness to text message outreach. A study with older patient populations in Italy found that vaccine passports influenced patient receptiveness to being vaccinated, as the vaccination card allowed them to access to public spaces [11]. Policies around vaccination status may have shaped patient decision-making regarding vaccine uptake in the United States as well.

### Implications of Study Findings

There are important lessons learned from this vaccination effort, which most health care systems undertook with no prior knowledge of how to execute this monumental task. The first one is the importance of accurate and updated patient contact information, particularly as it impacts effective outreach modalities. The second one is identifying patient preferences for communication to increase the likelihood of engagement. The third one is the need for interoperability of patient health records to triangulate patient touch points with other health systems and services. This ensures patient health records are up to date, and finite outreach resources are focused on priority groups. The final lesson is establishing strong relationships with neighboring health systems and government agencies for the purpose of coordinating outreach efforts and sharing pertinent patient data with each other. The COVID-19 vaccination campaign is still underway in the United States, and health systems need to be nimble and flexible in reaching out to patients, including nondigital efforts such as provider-patient communication, and partnering with community-based organizations to reach vulnerable patients.

### Limitations

There are several strengths and limitations to this study. A strength is the use of real-time patient data, which allowed capture and analysis of text outreach data linked to patient health records. This study provides critical data on digital outreach efforts as part of the COVID-19 vaccination campaign, which has important implications for local, regional, state, and national vaccination efforts. However, there are some limitations. First, 14,419 patients were excluded owing to shared phone numbers. The excluded patients had a similar demographic background as the study sample (Table 1). Second, it was not possible to verify if the patients received a COVID-19 vaccine outside of the study site.

### Conclusions

The United States has made significant strides in vaccinating Americans aged 65 years and older through a combination of digital outreach efforts, community-based vaccine clinics, and at-home visits. This study highlights the benefits and challenges of using text messaging outreach methods, specifically the ability to reach large numbers of patients quickly. While the majority of Americans aged 65 years and older have been fully vaccinated (87.1%-92.7%), younger Americans aged 18-39 years are lagging behind in vaccination rates [12,13]. Emerging data have shown disparities by geographic location with lower vaccination rates in rural areas and differences by patient demographics (eg, education level and political beliefs) [14,15]. Guidelines regarding booster vaccines to protect against new variants will require expanded and ongoing outreach efforts to eligible patients. Reaching these populations will require multiple outreach methods (eg, texting, email, phone calls, and community vaccine clinics) designed to address vaccine hesitancy and access issues. These last 2 points necessitate partnering with trusted figures in the community to encourage vaccination (eg, faith-based and community leaders), providers respectfully and empathetically answering patient questions about the vaccine, health care systems and medical institutions building relationships with community members, and making it easy and simple to get vaccinated through expanded vaccination locations with flexible operating hours [4]. Furthermore, sharing of patients’ vaccination statuses among health systems is integral to ensuring that outreach efforts are focused on unvaccinated individuals and individuals eligible for a booster. Coordination among health care systems, partnerships with and input from community leaders and members, and persistence are key elements to increasing COVID-19 vaccine uptake.

## Appendix

Multimedia Appendix 1 Sample demographics and interaction with text outreach (N=30,826).

## Abbreviations

OR: odds ratio
