# Supplementary material for: Evaluation of a Text Message–Based COVID-19 Vaccine Outreach Program Among Older Patients: Cross-sectional Study
Source: JMIR Form Res. 2022 Jul 18;6(7):e33260. doi: 10.2196/33260 (PMC9295725; doi:10.2196/33260)
Supplement: Multimedia Appendix 1 [file formative_v6i7e33260_app1.docx]

| **Multimedia Appendix 1: Sample Demographics & Interaction with Text Outreach (N=30,826)** | | | | | | | | | | | | | | | | |
| --- | --- | --- | --- | --- | --- | --- | --- | --- | --- | --- | --- | --- | --- | --- | --- | --- |
|  | **Total Sample** | **Location** | | **Race/Ethnicity** | | | | | **Age** | | | | | | **Sex** | |
|  |  | **Priority Zip Code** | **Non-Priority Zip Code** | **Black/AA** | **Hispanic** | **Asian** | **White** | **Other** | **65-69** | **70-74** | **75-79** | **80-84** | **85-89** | **90+** | **Female** | **Male** |
| **Patient Population** | 30,826 | 24,211 (78.5%) | 6,615 (21.5%) | 19,372 (62.8%) | 706 (2.3%) | 208 (.67%) | 7,315 (23.7%) | 3,225 (10.5%) | 9,804 (31.8%) | 7,770 (25.2%) | 5,151 (16.7%) | 3,504 (11.4%) | 2,273 (7.4%) | 2,324 (7.5%) | 18,399 (59.7%) | 12,427 (40.3%) |
| **Text Status** | | | | | | | | | | | | | | | | |
| **Delivered, Interested** | 2,938 (9.5%) | 2,225 (9.2%) | 713 (10.8%) | 1,782 (9.2%) | 36 (5.1%) | 24 (11.5%) | 787 (10.8%) | 309 (9.6%) | 1,351 (13.8%) | 909 (11.7%) | 401 (7.8%) | 171 (4.9%) | 68 (3.0%) | 38 (1.6%) | 1,741 (9.5%) | 1,197 (9.6%) |
| **Delivered, Not Interested** | 2,577 (8.4%) | 1,662 (6.9%) | 915 (13.8%) | 955 (5.1%) | 32 (4.5%) | 21 (10.1%) | 1,269 (17.3%) | 260 (8.1%) | 1,001 (10.2%) | 857 (11.0%) | 446 (8.7%) | 173 (4.9%) | 63 (2.8%) | 37 (1.6%) | 1,505 (8.2%) | 1,072 (8.6%) |
| **Delivered, No Response** | 12,333 (40%) | 10,083 (41.6%) | 2,250 (34.0%) | 7,951 (41.0%) | 484 (68.6%) | 100 (48.1%) | 2,223 (30.4%) | 1,575 (48.8%) | 5,296 (54.0%) | 3,335 (42.9%) | 1,831 (35.5%) | 1,058 (30.2%) | 453 (19.9%) | 360 (15.5%) | 6,178 (33.6%) | 6,155 (49.5%) |
| **Not Delivered** | 12,978 (42.1%) | 10,241 (42.3%) | 2,737 (41.4%) | 8,644 (44.6%) | 154 (21.8%) | 63 (30.3%) | 3,036 (41.5%) | 1,081 (33.5%) | 2,156 (22.0%) | 2,669 (34.4%) | 2,473 (48.0%) | 2,102 (60.0%) | 1,689 (74.3%) | 1,889 (81.3%) | 8,975 (48.8%) | 4,003 (32.2%) |
| **Preferred Communication Method** | | | | | | | | | | | | | | | | |
| **Phone Call** | 1,117 (38.8%) | 966 (43.4%) | 211 (29.6%) | 848 (47.6%) | 12 (33.3%) | 8 (33.3%) | 198 (25.2%) | 111 (35.9%) | 522 (38.6%) | 391 (43.0%) | 151 (37.7%) | 70 (40.9%) | 22 (32.4%) | 21 (55.3%) | 734 (42.2%) | 443 (37.0%) |
| **Email** | 834 (29%) | 635 (28.5%) | 199 (27.9%) | 505 (28.3%) | 13 (36.1%) | 13 (54.2%) | 208 (26.4%) | 95 (30.7%) | 427 (31.6%) | 240 (26.4%) | 101 (25.2%) | 32 (18.7%) | 21 (30.9%) | 13 (34.2%) | 476 (27.3%) | 358 (29.9%) |
| **Incomplete** | 927 (32.2%) | 624 (28.0%) | 303 (42.5%) | 429 (24.1%) | 11 (30.6%) | 3 (12.5%) | 381 (48.4%) | 103 (33.3%) | 402 (29.8%) | 278 (30.6%) | 149 (37.2%) | 69 (40.4%) | 25 (36.8%) | 4 (10.5%) | 531 (30.5%) | 396 (33.1%) |
| **Appointment Scheduling Status** | | | | | | | | | | | | | | | | |
| **Scheduled** | 226 (11.2%) | 188 (11.7%) | 38 (9.3%) | 185 (13.7%) | 1 (4.0%) | 1 (4.8%) | 23 (5.7%) | 16 (7.8%) | 97 (10.2%) | 86 (13.6%) | 29 (11.5%) | 5 (4.9%) | 2 (4.7%) | 7 (20.6%) | 143 (11.8%) | 83 (10.4%) |
| **Not Scheduled** | 1,785 (88.8%) | 1,413 (88.3%) | 372 (90.7%) | 1,168 (86.3%) | 24 (96.0%) | 20 (95.2%) | 383 (94.3%) | 190 (92.2%) | 852 (89.8%) | 545 (86.4%) | 223 (88.5%) | 97 (95.1%) | 41 (95.3%) | 27 (79.4%) | 1,067 (88.2%) | 718 (89.6%) |
| **First Appointment Status (among scheduled** | | | | | | | | | | | | | | | | |
| **First Appointment** | 214 (94.7%) | 178 (94.7%) | 36 (94.7%) | 175 (94.6%) | 1 (100%) | 1 (100%) | 23 (100%) | 14 (87.5%) | 93 (95.9%) | 80 (93.0%) | 28 (96.6%) | 5 (100%) | 2 (100%) | 6 (100%) | 135 (94.4%) | 79 (95.2%) |
| **Not Completed** | 12 (5.3%) | 10 (5.3%) | 2 (5.3%) | 10 (5.4%) | 0 (0.0%) | 0 (0.0%) | 0 (0.0%) | 2 (12.5%) | 4 (4.1%) | 6 (7.0%) | 1 (3.4%) | 00 (0.0%) | 0 (0.0%) | 0 (0.0%) | 8 (5.6%) | 4 (4.8%) |
| **Second Appointment Status (among scheduled** | | | | | | | | | | | | | | | | |
| **Second Appointment** | 207 (96.7%) | 171 (96.1%) | 36 (100%) | 168 (96.0%) | 1 (100%) | 1 (100%) | 23 (100%) | 14 (100%) | 90 (96.8%) | 77 (96.3%) | 27 (96.4%) | 5 (100%) | 2 (100%) | 6 (100%) | 129 (95.6%) | 78 (98.7%) |
| **Not Completed** | 7 (3.3%) | 7 (3.9%) | 0 (0.0%) | 7 (4.0%) | 0 (0.0%) | 0 (0.0%) | 0 (0.0% | 0 (0.0%) | 3 (3.2%) | 3 (3.8%) | 1 (3.6%) | 0 (0.0%) | 0 (0.0%) | 0 (0.0%) | 6 (4.4%) | 1 (1.3%) |
